# SITHON: An Airborne Fire Detection System Compliant with Operational Tactical Requirements

**DOI:** 10.3390/s90201204

**Published:** 2009-02-24

**Authors:** Charalabos Kontoes, Iphigenia Keramitsoglou, Nicolaos Sifakis, Pavlos Konstantinidis

**Affiliations:** 1 Institute for Space Applications and Remote Sensing, National Observatory of Athens / Metaxa & Vassileos Pavlou Str, GR 152 36, Palea Penteli, Athens, Greece; E-Mails: ik@space.noa.gr (I.K.); sifakis@space.noa.gr (N.S.); 2 Forest Research Institute, National Agricultural Research Foundation, 570 06 Vassilika, Thessaloniki, Greece; E-Mail: pavkon@fri.gr

**Keywords:** Wildland fires, thermal infrared uncooled camera, airborne imaging system, Inertial Navigation System (INS), Global Positioning System (GPS)

## Abstract

In response to the urging need of fire managers for timely information on fire location and extent, the SITHON system was developed. SITHON is a fully digital thermal imaging system, integrating INS/GPS and a digital camera, designed to provide timely positioned and projected thermal images and video data streams rapidly integrated in the GIS operated by Crisis Control Centres. This article presents in detail the hardware and software components of SITHON, and demonstrates the first encouraging results of test flights over the Sithonia Peninsula in Northern Greece. It is envisaged that the SITHON system will be soon operated onboard various airborne platforms including fire brigade airplanes and helicopters as well as on UAV platforms owned and operated by the Greek Air Forces.

## 1. Introduction

Timely digital geo-referenced fire location maps are essential for tactical fire intelligence and mapping. Fast fire detection and real time monitoring of fire parameters, such as fire spread and location of hot spots, are crucial for the success of the initial attack on and containment of a fire. For this purpose, thermal infrared sensors and airborne infrared line scanners or frame cameras should be regarded as components of such a detection and monitoring system. These can provide fundamental information on fire extent and intensity, as well as rate of progress, which are of vital importance for tactical suppression actions.

Information is required at different levels, and should be customised to user needs. For instance, at a *strategic* level, information on the fuel load, the assets and land cover distribution at different regions is needed to allocate national and regional priorities and to support decisions such as the regional distribution of budgets, personnel and equipment as well as to establish and maintain initial attack capabilities, and weather and fire monitoring systems. At the same time, at a *management* level, information is needed to support fire occurrence monitoring, fire behaviour process models and fire occurrence prediction systems. Finally, at an *operational* level, especially during a crisis, tactical observations are those that provide detailed, frequent, and repetitive views of specific fire events. Added-value products from these observations include the fire perimeter, fire front, and isolated hot spots and they are used to support decision making for initial attack response, logistical and tactical planning and sustained action operations [1]. However, these different levels have very different requirements in terms of spatial and temporal resolution. Remote sensing systems can provide information at all levels. In the case of tactical emergency response, these systems can be mounted on fixed, airborne or satellite platforms. These are summarized hereafter.

### 1.1. Remote Sensing Systems for Wildfire Emergency Management

Automatic detection systems mounted on *fixed platforms* can make use of cameras on towers, buildings or masts with good visibility of the surveyed terrain. The sensor technologies used in today's automatic ground detection systems are mainly infrared cameras, but in some cases optical cameras may be used. Autonomous detection of hot spots in the thermal infrared (TIR) part of the electromagnetic (E/M) spectrum is the basis for several commercial systems, such as BOSQUE (IZAR-FABA) and BSDS (Teletron). The images are sent to a central control unit via a radio communication system [2, 3].

Major fires are detectable from space and their progress can be monitored using *satellite* technology. Satellite systems are by far the mostly developed fire remote sensing tools. Satellites providing information in the infrared spectrum by deploying TIR imaging spectrometers are suitable for fire detection. Satellites and sensors with such capabilities include MSG-SEVIRI and GOES, NOAA –AVHRR, TRMM-VIRS, METOP-AVHRR-3, TERRA-MODIS, AQUA-MODIS, ERS2-ATSR, ENVISAT-AATSR, LANDSAT-TM/ETM+ and TERRA-ASTER. However, none of them has been used operationally for fire detection, mainly due to insufficient revisiting time (with the exception of MSG-SEVIRI and GOES, which are on a geostationary orbit and provide images every 15 minutes, however, with a coarse spatial resolution on the ground). It is noteworthy that these satellites can be used in a multi-sensor approach to improve the temporal resolution and may also be used in a complementary way with other surveillance systems. Near real time monitoring to support civil protection services as well as regional and national authorities has been implemented in the framework of RISK-EOS, a service element within the Global Monitoring for Environment and Security (GMES) program funded by European Space Agency and the European Commission.

Apart from the operational satellites, whose primary task, however, is not fire detection, there are plans for certain satellite missions dedicated to fire detection. For instance, FUEGO [4] is planned to be a satellite system intended to provide the fire fighting forces with early fire warning and fire monitoring capabilities at selected regions of the Earth which are considered at high fire risk. Each satellite will have a payload including a sensor that will acquire information in the middle infrared region of the spectrum, in addition to dedicated thermal sensors designed especially for the detection of fires. Since the temporal resolution is the primary mission requirement and the number of satellites must be kept as low as possible for economic reasons, the orbital configuration has exceptional importance. If this mission succeeds, it will be the first one specifically dedicated to fire detection. It has to be stressed though that cost-effectiveness of satellite networks strictly dedicated to fire detection is somewhat questionable. Another innovative initiative reported is that of the DLR (German Aerospace Agency), which participates in the development of new satellites and sensors for fire detection. The Bispectral Infra-Red Detection (BIRD) and an Intelligent Infrared Sensor System, called FOCUS have the objective of testing a new generation of infrared array sensors adapted to Earth remote sensing objectives by means of small satellites. BIRD, which was launched in October 2001, incorporates thematic on-board data processing through a neural network classifier, and real-time discrimination between smoke and water clouds. BIRD consists of the Hot Spot Recognition System (HSRS) and the Wide-Angle Optoelectronic Stereo Scanner (WAOSS-B) [5]. Finally, the FOCUS sensor, based on the same principles as BIRD, is to be mounted on the International Space Station (ISS) [2].

The fixed orbit of satellite platforms applies a restraint in revisiting capability both in tactical operations during the crisis that require frequent and repetitive observations, and in surveillance of vulnerable areas under high-risk conditions. *Aircraft* (manned or unmanned) are manoeuvrable and may very quickly revisit the critical areas providing rapid response for emergency situations. Airborne TIR sensors (usually cameras) can detect new hot spots that develop rapidly into wildland fires. Besides aircrafts equipped with TIR sensors can be used for supporting fire-fighters in safety tasks, and for detecting escape routes or security zones, in areas where the human visibility is restricted due to the smoke.

In the 1980s the first research and development activities on infrared fire imaging from aircraft started at the National Interagency Fire Center (NIFC). These activities included the use of forward looking infrared (FLIR) systems [6], and development of the so-called FIRE MOUSE TRAP imaging system (Flying Infrared Enhanced Maneuverable Operational User Simple Electronic Tactical Reconnaissance And Patrol, see e.g. [7]). Subsequently, the Forest Service and NASA-JPL (Jet Propulsion Laboratory) jointly developed the FIREFLY thermal fire imaging system, which was the basis for the subsequent PHOENIX onboard a Cessna Citation Bravo jet and a King Air 200 turboprop. During the operation of these airborne cameras the pressing need was realised for an integrated system that would transmit temperature information to the ground in real time. To meet this need, NASA-Ames Research Center first developed the Airborne Infrared Disaster Assessment System (AIRDAS), which filled a critical gap in remote sensing fire research supporting the fire combating operations. The following years AIRDAS was further developed to include improved telemetry, rapid geo-referencing algorithms, and multiple spectral band data collection and transmission. These led to the unmanned aerial vehicle fire response (UAV FiRE) project in 2001 [8].

Other systems used in different countries incorporating thermal infrared technology to assist forest fires fighting include surveillance and co-ordination airplane ACO (DGCN Dirección General de Conservación de la Naturaleza belonging to the Ministry of Environment of Spain), Daedalus System (Daedalus Enterprises), Wildfire Airborne Sensor Programme WASP (Rochester Technology Institute) and Airborne Wildfire Intelligence System AWIS (Range and Bearing Environmental Resource Mapping Corporation). A review of airborne infrared systems is conducted by Rodriguez Y Silva [3].

This brief review proves that in the last decade there have been advancements in technologies supporting wild land fire management and tactical emergency response [8, 9, 10]. These advancements include: a) improved capability for remote sensing towers, aircrafts and satellites; b) Geographic Information Systems and Decision Support Systems [11]; c) image processing and image geo-referencing; d) Global Positioning System (GPS) in combination with Inertial Navigation Systems (INS); and e) fire behaviour models [12, 13]. In addition, uncooled forward-looking infrared scanners and cameras provide an opportunity for lower cost fire detection and monitoring systems.

The present paper introduces SITHON, a complete system tailored to the unique requirements of the wildland fire fighting community. Section 2 presents the system in detail, whilst Section 3 demonstrates the results of a test flight. In Section 4 the main conclusions are drawn.

## 2. SITHON

### 2.1. SITHON project Overview

The SITHON Project was funded in 2003 under the Operational Program of Competitiveness of the Greek Ministry of Development-General Secretariat for Research and Technology (Action 4.5.1: Natural Environment and Sustainable Development). The project lasted three years and was initiated in July 2003. The main objectives of the project were to foster collaboration between the private service sector, technological partners and research community, to demonstrate the synergy of evolving technologies (both terrestrial and airborne) for increasing the information content of data collected, and enhancing the timeliness in data collection, processing and transition towards an effective detection, monitoring and management of wildfire suppression actions.

It is well known that time is a crucial parameter in fire combating and fire containment. The level of efficiency depends on the availability of fire fighting resources, the firemen endurance, as well as the knowledge of the terrain, the fire location and the ways to access to it. For this purpose the SITHON project applied remote sensing systems of terrestrial and airborne detection sensors for the localization, notification and monitoring of the active forest fires. The whole SITHON system comprises a wireless network of *in-situ* optical cameras, and an airborne fire detection system based on a fully digital thermal imaging sensor. The network of sensors is linked to an integrated GIS environment in order to facilitate the fire fighting management and support the decision making process during crisis. The GIS data bases incorporates qualitative and quantitative information layers needed to estimate the fire risk, such as the vegetation types and quantity of fuel matter, the road network for accessing the active fires, the area's morphology, the sensitive and endangered locations (settlements, camps, folds, archaeological sites, etc), the high danger infrastructures (fuel stations, flammable materials, industrial areas, etc), the natural or artificial water reservoirs, etc. The platform of SITHON includes a Crisis Operating Centre, which receives information in the form of images and data from the wireless sensor detection systems, displays it on wide screen monitors and analyses it to derive the dynamic picture of fire evolution. In the following sections a detail description of the airborne thermal imaging part of the SITHON system is given.

### 2.2. Goals and Specifications of the airborne thermal imaging system

The SITHON project aimed at conducting further R&D in the area of using new smaller and less expensive IR uncooled cameras for fire detection and monitoring purposes, and post-fire management (i.e., mapping of burnt areas, assessment of fire impact). SITHON also focused in the area of image processing coupled with Geographic Information System (GIS), Global Positioning System (GPS) and Inertial Navigation System (INS) technologies to assist in the dynamic translation of the raw image data collected (video outputs or thermal images) to usable products. The SITHON airborne system was designed to offer high quality colour (video RGB) and infrared imagery. Each frame acquisition is coupled with kinematic GPS and inertial platform data ingested by a precise MIDG II Inertial Measuring Unit (IMU) on board the airplane. The integration of the GPS, the IMU and camera data along with a digital elevation model (DEM) allows for real time orthorectification and geo-positioning of the acquired frames without using ground surveyed control points.

The ultimate aims in SITHON system design were directed to meet operational tactical requirements and products generation which are useful for crisis management including:

- hot spot identification, geo-positioning and alarm generation,
- fire front location and representation on a map,
- isothermal contour lines generation and mapping,
- geo-referenced video (RGB) and thermal image frames projected to the Hellenic Geodetic Reference System (EGSA87) or any other cartographic projection system used by the fire managers in the area of interest,
- synthetic cartographic products integrating georeferenced thermal and/or video images with existing background maps of assets, transportation networks and facilities situated in the area of operations.

**Figure 1.**
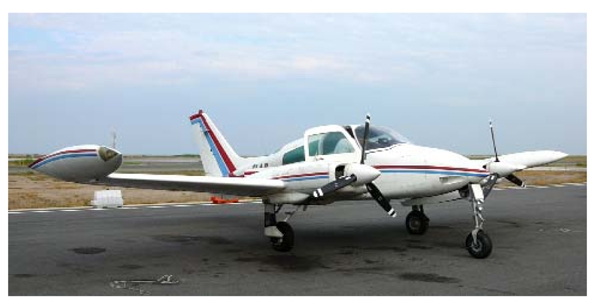
The CESSNA 310Q two-engined aircraft which is the platform of SITHON

Timeliness is of paramount importance for the success of such a system. The system is designed to ensure automatic fire detection. It is mountable on any airborne platform and can be operated within 15 to 20 minutes after the first fire announcement. Once on the platform, SITHON is supported by a fully automated control system, which manages the frame acquisition, the radiometric image calibration and thresholding, as well as the dynamic hot spot/fire front identification and geo-positioning within 50-100 m error with the lack of any operating GPS station on the ground. To be noted that the off-line processing of stereo pairs of images with the incorporation of GPS ground reference data, improves the geo-positioning accuracy up to the order of 1-3 m (∼1,5-2 pixels). Clearly, the post-processing of stereo pairs of images is useful only for post-crisis management to allow precise mapping and assessment of the damages in the impacted zones. During crisis, the imaging system is integrated with telemetry equipment ready to transmit the output products to fire-fighting forces in real-time. The platform of SITHON was a CESSNA 310Q two-engine aircraft own and operated by AEROPHOTO Ltd (Figure 1).

### 2.3. Imaging payload

#### 2.3.1. Camera

The integration of the camera was simplified due to existing design elements of the plane. For instance, the aircraft was equipped with a gyro-stabilized camera mount, on which the infrared camera body was mounted and fixed. The Thermovision R 570 camera used is a light-weight high performance uncooled forward looking infrared camera. It uses advanced uncooled Focal Plane Array (FPA) micro-bolometer technology and stores images and data to memory cards. The detector consists of a cavity with 77 760 microbolometers made of Si-elements coated with a thermal resistance layer. The microbolometers are thermal detectors and are operated without cooling. But besides the thermal radiance of the target, the radiation of internal camera components may be detected by the uncooled detector arrays, and this can decrease the measurement accuracy. To tackle this problem the inside case temperature is measured at several locations and these measurements are used in the automatic re-calibration of the camera at predefined time intervals. In addition, in order to avoid any convective heat transfer to the detector elements, the bolometers are placed in a vacuum chamber and their temperature is kept close to the ambient temperature using Peltier elements. Although the response time of bolometers is remarkably longer than quantum detectors, an image frequency of 60 Hz is achievable with this system. The absence of moving parts makes the operation of the Thermovision R 570 IR-camera of AGEMA more comfortable and safe. Bolometers are not selective with respect to the spectral range, therefore optical filters are used with this camera to optimise response at a spectral range of 7.5-13 μm. The camera optical system is using lenses with a focal length (*f*) of 20 mm.

The technical characteristics of Thermovision R570 infrared camera are summarized in Table 1. The camera has been calibrated in the laboratory to resolve fire intensities up to 500 °C. For this range of temperatures discrimination was approximately 1.0 °C per digital count with a systematic shift (bias) of 6 °C. This was considered effective for the purposes of SITHON project as the interest was focused on mapping relative temperature differences between points rather than estimating absolute temperatures.

**Figure 2.**
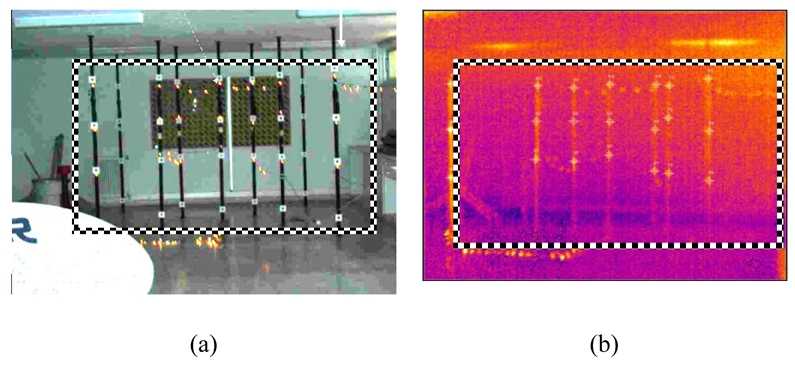
(a) 3-D calibration tests deployed in the Photogrammetry Laboratory (NTUA) and (b) a sample image taken during the calibration test. The black and white box indicates the effective area of measurements.

In order to recover precisely the camera's internal characteristics, that are the coordinates of the principal point *(x_p_, y_p_)* and the principal distance *(c)*, the camera underwent an analytical 3-D calibration (bundle adjustment with self calibration), using the 3-D calibration test field of the Laboratory of Photogrammetry [shown in Figure 2(a)] belonging to the School of Rural & Surveying Engineering (National Technical University of Athens). The resulted interior camera orientation after extensive tests [an example is shown in Figure 2(b)], is reported in Table 1. In order to facilitate the image ortho-rectification calculations during the operations, the interior orientation parameters *x_p_, y_p_* and camera constant *c*, have been expressed in pixel units with respect to FPA dimensions (320×240 pixels). The best value for the calibrated camera constant *c* was estimated to be 763,871 pixels, which is equivalent to a focal length of 19,86 mm, given that the physical dimension of pixel is 26 μm. This calibration procedure is repeated annually prior to fire season and camera deployment.

The forward motion of the aircraft during camera operation may result in a blurring effect of the acquired image. The image blur parameter is defined as:

(1) $$\text{blur}=V\;\frac{f}{H}\;\Delta t\;\cos\theta$$

where *V* is the velocity of the platform (aircraft), *f* is the camera focal length, *H* is the flight height above ground level, Δ*t* is the time for exposure and *θ* is the off nadir angle. The SITHON imaging system has been designed as to avoid blurring effects on the acquired images. Taking as example the height and aircraft velocity conditions during SITHON demonstration, mounted on a CESSNA 310Q airplane flying at 160 km/h and 7500 ft (∼2300 m) above the ground, and considering that the camera is looking at nadir (θ= ∼ 0°) with lenses of 19,86 mm focal length and exposure time of 1/60 sec, the blur parameter is estimated to be of 0,006 mm or 6μm. Therefore, the effect of blur is about the 1/5 of pixel's physical dimension, which is not discernible by human eye. It should be noted that the FPA detector size of the Thermovision R 570 IR-camera of AGEMA is 0.026 mm or 26μm (AGEMA 570 Operating Manual). Table 2 shows the variation of the blur parameter for different flying heights and aircraft velocities during the demonstration of the SITHON airborne system. In all cases the estimated size of the forward motion blur is less than the FPA pixel size. In addition, Table 2 shows the ground surface detected by the SITHON imaging sensor for combinations of flying altitude and aircraft velocities reported during the system demonstration.

#### 2.3.2. Inertial measuring unit for positioning and orientation

**Figure 3.**
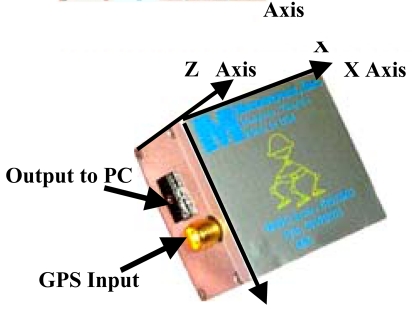
The MIDG II GPS/INS device of Microbotics.

As mentioned above, the direct geo-referencing of the acquired images is based on the combination of GPS measurements with inertial measurements for the camera orientation angles *(pitch, roll, and yaw)*. The IMU device operated by the SITHON system is the MIDG II of Microbotics (Figure 3 and Table 3).

It is a low-cost, light weight, small size and low power package which integrates an internal GPS receiver that collects GPS positions and velocity information and passes it to the data fusion processor to be combined with the inertial data to generate the state vector. During operations the device provides in real time differential measurements of linear accelerations and rotational rate increments. The actual position, velocity and platform attitude in relevance to an inertial coordinate system is obtained by integration of the dynamic differential recordings. Gyro and accelerometer data from the IMU device allow the accurate determination of both attitude *(pitch, roll, and yaw)* and position *(X_o_, Y_o_, Z_o_)* for the camera projection center at the time of exposure. To account for inaccuracies derived by device drifts in long time operations, the MIDG II inertial system is using a GPS receiver, which is ideally coupled with the inertial data using a Kalman Filter. This allows accurate upgrading of the estimated navigation and positioning data [9].

#### 2.3.3. Acquisition control system

**Figure 4.**
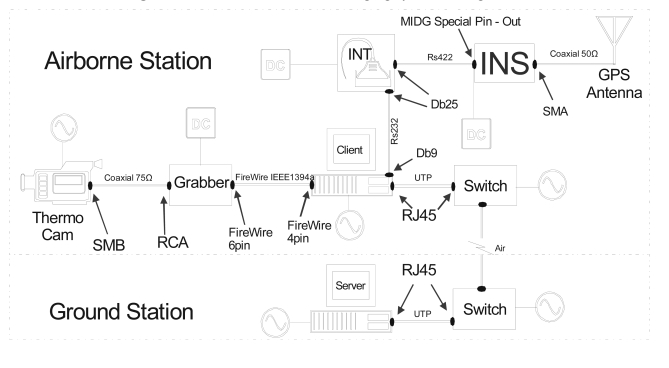
The SITHON airborne imaging system configuration.

Significant software developments were made to allow remote control and operation of the camera system by the payload engineer on board. The SITHON system configuration (airborne and ground subsystems) is shown in Figure 4.

**Figure 5.**
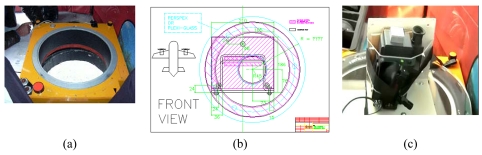
(a) Camera opening at the bottom of the aircraft CESSNA 310Q using an inertial gyroscopic platform GSM300, (b) Design for camera fit to the camera opening at the bottom of the aircraft, (c) Camera and IMU devices rigidly attached.

The payload components (camera, GPS and INS units) are controlled by a laptop PC (Pentium IV, 2GHz) onboard the airplane. The camera and the IMU are rigidly attached and oriented to the camera opening at the bottom of the aircraft (Figure 5). The system commands the acquisition of images at the appropriate time intervals, accounting for the aircraft velocity, the flying height and the physical camera frame dimensions, in order to acquire imagery without gaps in terrain coverage and to allow adequate overlapping along the flight axis in case stereoscopic acquisition is required for off-line processing. This operation is fully automatic and is based on the following formula:

(2) $$\text{Time betweensuccessive acquisitions}=\frac{H}{f}\frac{l}{V}\;\;(1-OV)$$

where *l* is the FPA physical dimension (mm) along the flight axis (*l* = 6.24 mm), *V* is the velocity of aircraft, *f* is the camera focal length, *H* is the flight height above ground level, and *OV* is the percentage overlap, which equals to 0% for monoscopic view or at least 60% for a stereoscopic one. Table 4 illustrates the time interval of successive image acquisitions, which have been tested during the demonstration of SITHON system.

During the operations the payload engineer is provided with several interfaces showing dynamically (i) the status of system's components and subcomponents, (ii) the data input to the system (images, GPS and INS data), (iii) the status of communication between the system controller (onboard) and data server (on the ground), and (iv) the output products (geo-referenced image, geographic coordinates of the hot spots detected, the id of the data package processed, etc).

#### 2.3.4. Image processing

For every camera exposure the corresponding GPS and IMU data are automatically registered together with the image data to create a new *data package*. The data package is forwarded to a server PC system (Pentium IV, 2GHz), installed on the ground station or on board the aircraft (depending on the chosen communication architecture). The *data package* is decoded on the server PC, and transformed to useful products before the next camera frame is captured. The following operations are carried out on the server station in real time:

- image geo-rectification and projection,
- identification of temperature alarms (image processing),
- display of the geographic coordinates of detected hot spots and fire fronts.

Direct image geo-referencing is based on a) the combination of the GPS data for the positioning of the camera exposure centre, and b) the inertial measurements returning the camera's orientation angles at the time of exposure. The restored and ortho-rectified thermal or video images are projected to the reference cartographic projection system, and undergo a number of additional processing operations including global image thresholding, in order to detect areas with temperatures exceeding a predetermined value (hot spots). The generated temperature and hot spots map products are then combined with existing background and asset maps of the area under monitoring.

**Figure 6.**
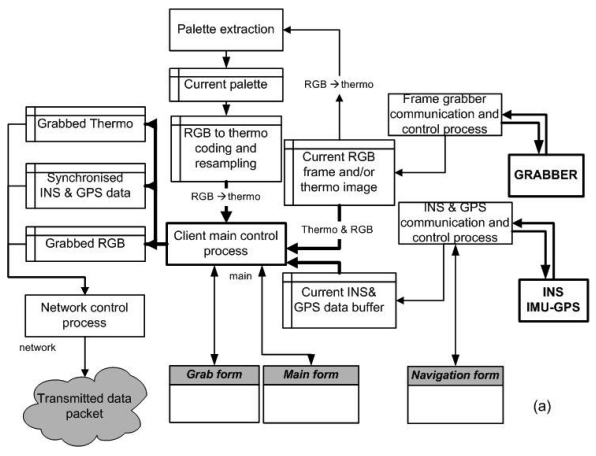

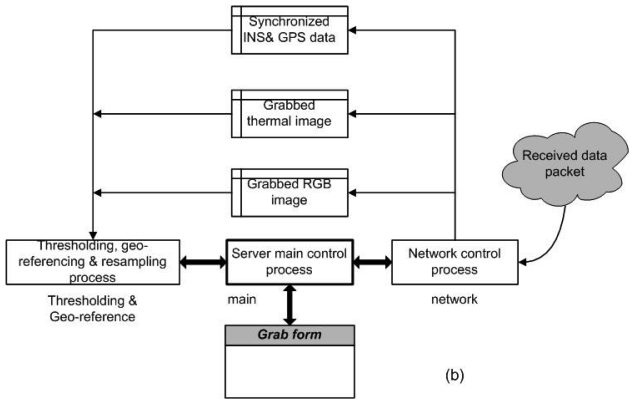
(a) S/W operations for sensor control and input raw data processing; (b) operations applied on the server PC station for data package decoding, image thresholding, and image geo-referencing.

Figure 6(a) illustrates the set of operations undertaken by the software package, which has been developed for sensor control and input raw data processing to derive a set of usable information products. Moreover, Figure 6(b) summarizes the operations applied on the server PC station including data package decoding, image thresholding and geo-referencing.

## 3. Demonstration test flight

**Figure 7.**
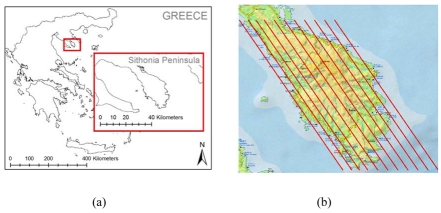
(a) The Sithonia peninsula study area over which the SITHON system demonstrations were carried out. (b) Pre-designed navigation paths inserted to aircraft's navigation control system for acquiring imagery with no gaps in terrain coverage.

Several demonstrations of the SITHON airborne imaging platform have been conducted during the project lifecycle. The first experiments were carried out in May 2005. During these operations new requirements for additional R&D developments were identified for enhancing system's capabilities meeting operational functionality needs. The system in its final configuration was flown over the Sithonia Peninsula of Chalkidiki in May 2006 [Figure 7(a)]. The system's sensing and imaging capability was evaluated in different conditions by changing the flight height and aircraft cruising speed, as well as the size and the intensity of fire flames detected. For this to be achieved, a number of different pre-designed navigation paths as in Figure 7(b), have been inserted into the aircrafts navigation control system to ensure that the entire Sithonia Peninsula is covered without gaps in image acquisition.

During the demonstration campaigns the entire study area was surveyed by the SITHON system by capturing and processing acquisitions every 5-7 seconds. The exact time interval between successive acquisitions depended on the flying conditions (flight height, cruising speed, ground elevation). The computer system controlling the image capture during the flight was calculating and automatically adjusting the time interval between the successive frames. For as long as the level of recorded temperatures was within ordinary values the system was remaining in the so-called ‘silent mode’ sending to the server PC every processed and geo-referenced thermal image frame.

**Figure 8.**
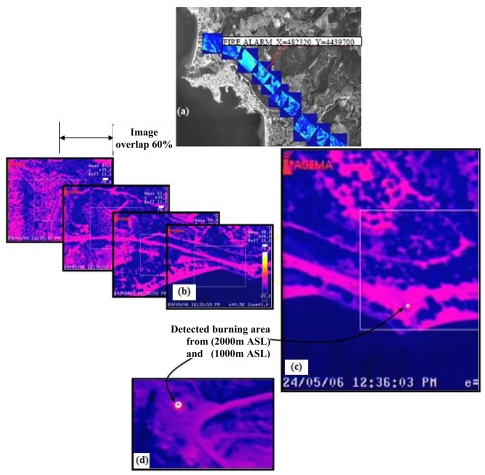
The operation of the SITHON airborne imaging system on May 24, 2006 over the Sithonia peninsula: (a) and (b) dynamic surface viewing and image stereoscopic acquisition with 60% image overlap, (c) a 3×3 m^2^ burning surface detected from 2,000 m ASL and (d) a 3×3 m^2^ burning surface detected from 1,000 m ASL

As long as the first hot spot event with a temperature above the predefined threshold limit was detected, the system turned immediately to ‘alarm mode’ and the aircraft staff was alerted to the danger by a sound alarm. At the same time, the geographic coordinates of the fire event were shown on the display, indicating the existence and location of the fire as in Figure 8. The demonstration campaigns showed that the SITHON infrared platform has been sensitive enough to detect fires of low intensity and of a few square meters on the ground from the height of 1,000 m to 2,000 m above sea level (ASL). Real time direct image geo-referencing, using input only from the GPS-assisted INS, performed well and produced sufficiently accurate (∼50 m) relationships between the captured images and the terrain system. Figures 8(a) and (b) illustrate the system's capability for dynamic surface representation, stereoscopic viewing with image overlap of 60% and image projection of the geo-referenced images onto a reference base map of the study area. An example of a low intensity and small size fire of about 3×3 m^2^ on the ground as detected from 2,000 m and 1,000 m ASL is given in Figure 8(c) and (d), respectively.

## 4. Conclusions

The design and operation of SITHON, a fully digital airborne thermal imaging system integrating INS/GPS and a digital camera has been described and its demonstration results have been discussed. During the operations it was demonstrated the system's configuration robustness for secure processing and provision of results in real time while it is in the air, supporting the fire managers and fire fighters. Yet, most of the airborne platforms are mainly being used to drop water and retardant on top of fires. The present developments allow the command and operations control centre to pinpoint the path of the airborne sensor on a map and depict the evolution on the fire fronts during fire fighting. During system development the motivation behind engineering design was to meet the need of fire managers to follow in detail the magnitude of emergency, and also to run battling scenario online. For this purpose timely positioned and projected thermal images as well as video data streams rapidly integrated in the GIS systems operated by the Crisis Control Centres have to be available in an efficient manner. These issues were well solved and demonstrated by operating SITHON.

Some other issues still lay on the data link communication, which needs to be solved independently from place to place, as the legislation framework, and bandwidth permissions for establishing adequate air-ground data links between the airborne platform and the Crisis Control Centres vary with the country/region. Another remaining issue is the integration of cartographic projection systems, which are used in the various countries/regions wishing to operate the system. It is considered that both issues are easily resolvable in collaboration with the Authorities wishing to operate the system, by licensing the deployment of common wireless air-ground communication and providing access to the full set of their cartographic projection systems used (e.g., information on geoid, ellipsoid and cartographic projection used).

The conducted experiments have also shown that ortho-rectification in the air for real-time transfer of thermal images to the ground does not require any precise and fine resolution elevation model (DEM) of the observed area, considering also the rough positioning accuracy of the thermal sensor (∼50 m) provided by the single use of the camera GPS receiver. If this operation mode is selected, the use of a globally available DEM such as the SRTM data with a cell size of 90 m and cell height accuracy of ∼10-15 m is considered sufficient. However, if off-line stereo-pair image processing is requested for burn scar mapping and damage assessment using data from a GPS base station operated on the ground, then it is of utmost importance to use high quality DEMs in order to obtain the appropriate mapping accuracies.

Today the disaster management and social security community can profit from the technological advancements, enabled by the combined use of image processing, navigation, and Earth Observation technologies, with the existing and planned sensor systems of high observing sensitivity, and airborne platforms in particular UAVs. For these purposes it is envisaged that the SITHON system will be operated onboard multitude airborne platforms including the fire brigade airplanes and helicopters as well as the UAV platforms owned and operated by the Greek Air Forces. In this context the authors are in discussion with the Civil Protection and Air Forces authorities in Greece and other south European Mediterranean countries, for flying the SITHON airborne system during the crisis situations imposed by wildfires and flooding. Through national and European projects the authors are committed to assisting and improving the development of these supporting technologies so that they are operationally used by the disaster management community.

## Figures and Tables

**Table 1.** AGGA Thermovision R570 Infrared camera characteristics

FOV (field of view)	24°×18°/0.5 m built in
IFOV (instantaneous field of view)	1.3 mrad
Detector type	Focal Plane Array / uncooled Microbolometer 320×240pixels, physical pixel dimension = 26 μm
Spectral range	7.5-13.0 μm
Object temperature measurement range	-20 °C to 500 °C (-4 °F to 930 °F) up to +1500 °C (+2700 °F), with high temperature option
Measurement accuracy	± 2 % of range or ± 2 °C
Thermal sensitivity	< 0.15 °C
Video output	CCIR/PAL Composite and S-video
Image radiometric resolution	12 bit images stored on PC-Card hard disc
Camera platform interior orientation parameters (in pixels/ principal point *x_p_, y_p_* and camera constant *c*)	x_o_=16,0452, y_o_=31,6923,c=763,871 (equivalent to 19,86 mm focal length)

**Table 2.** SITHON forward motion blur for different combinations of flying velocity and height (f=19,86 mm, FPA detector size=26 μm, Δt=1/60 sec).

**Flying velocity V (km/h)**	**Flying height H (m)**	**Blur (μm)**	**% FPA pixel size**	**Area detected on the ground L×W (m×m)**
160	1,000	14.8	57%	416 × 312
200	1,000	18.5	71%	416 × 312
160	1,500	9.90	38%	624 × 468
200	1,500	12.3	47%	624 × 468
160	2,000	7.40	28%	832 × 624
200	2,000	9.30	36%	832 × 624

**Table 3.** MIDG II Inertial Navigation Device characteristics

IMU Physical dimensions	3.8 × 2.2 × 4.25 cm
IMU Weight	55 gr
IMU Input Voltage	10VCD-32VDC
IMU Power	1,2 W max
GPS antenna connector type	50 Ohm SMA
GPS antenna power	+5 Volt at centre conductor, 25 ma max
GPS RF power input	-134 dBm min, -61dBm max
Measurements	
Attitude accuracy (roll, pitch, and yaw)	0.4 °
Position accuracy	3 m CEP, WAAS/EGNOS available
Data output rates	Position 10Hz, Velocity, attitude, acceleration 50Hz

**Table 4.** Time interval of successive image acquisitions for different combinations of flying velocity, flying height and image overlap percentage. FPA physical dimension *l* = (6,24mm) × *w* (8,32mm).

**Flying velocity *V* [km/h]**	**Flying height *H* [m]**	**Time interval in seconds - stereoscopic view (overlap 60%)**	**Time interval in seconds - monoscopic view (overlap 0%)**

160	1,000	3	7
200	*1,000*	2	6
160	1,500	4	11
200	1,500	3	8
160	2,000	6	14
200	2,000	4	11
